# ﻿*Paragomphus alami* sp. nov. (Odonata, Gomphidae): a new dragonfly species described from the White Nile River, Sudan

**DOI:** 10.3897/zookeys.1265.168108

**Published:** 2025-12-30

**Authors:** Mohamed Salah, Rania Baleela, Esraa Yousif Ahmed, Babiker Isam, Almontasirbillah Abdalla, Mai Masri, Esra Elfaki

**Affiliations:** 1 Sudan Nature Guide Project, Khartoum, Sudan; 2 Toxic Organisms Research Centre, Faculty of Science, University of Khartoum, Khartoum, Sudan; 3 Department of Zoology, Faculty of Science, University of Khartoum, Khartoum, Sudan; 4 Barcode Sudanese Organisms Project (BSOP), Khartoum, Sudan; 5 Environment, Natural Resources and Desertification Research Institute, National Center for Research (NCR), Khartoum, Sudan

**Keywords:** Biodiversity, COI barcoding, conservation, floodplain, Khartoum

## Abstract

Sudan’s unique biogeographic position at the Afrotropical-Palearctic interface, coupled with the ecological gradient of the Nile River, fosters a diverse odonate fauna. Despite this, the genus *Paragomphus* Cowley, 1934 remains understudied in the region. This study describes *Paragomphus alami***sp. nov.**, a new species of *Paragomphus* from the White Nile floodplain in Sudan, based on integrated morphological and molecular evidence. Field surveys conducted between 2017 and 2022 documented adult populations across the Sudanese floodplains. Specimens were morphologically analysed using microscopy compared to congeners *P. lacustris* Karsch, 1890 and *P. elpidius* Ris, 1921. DNA barcoding (COI gene) was performed on two specimens, with maximum-likelihood phylogenetic reconstruction using 28 sequences of *Paragomphus* and related species in addition to an outgroup. Mean interspecific genetic distance was computed manually. Morphological comparisons with congeners revealed unique diagnostic traits in *P. alami***sp. nov.**, including short, thick cerci ending with a black tooth, and an epiproct that is noticeably shorter than those of *P. lacustris* and *P. elpidius*. The phylogenetic analysis revealed that *P. alami* forms a well-supported monophyletic clade (bootstrap value = 100%), which is corroborated by morphological evidence, and no observed intraspecific variation, which supports the recognition of this species as distinct; this was further supported by the mean interspecific distance of 12.34%. This discovery highlights Sudan’s role as a biogeographic crossroads and the need for further research of Odonata in the region. Habitat sensitivity highlights conservation urgency. The species seasonal emergence, habitat specificity, and sensitivity to deforestation underscore its conservation importance.

## ﻿Introduction

Sudan lies at the intersection of the Afrotropical and Palearctic biogeographic regions and encompasses diverse habitat types ranging from arid deserts in the north to high-rainfall savannas in the south (Darbyshire et al. 2015). Influenced by climatic variability and the ecological gradient created by the River Nile, which traverses multiple ecosystems as it flows north, numerous insect species have dispersed beyond their Afrotropical origins. As a result, the Odonata of Sudan represent a biogeographic blend of Afrotropical and Palearctic elements. However, the odonate species richness in the Nile Basin remains comparatively low, with approximately 250 recorded species of the 900 estimated for Africa (slightly over 800 species according to Dijkstra (2007)). This contrasts with the Congo Basin where more than 40% of all African odonate species are concentrated (Dumont 2009). In Sudan, several odonate species appear to be geographically and genetically isolated from their counterparts in tropical Africa, as evidenced by notable morphological distinctions (Dumont 1988). The most recent published checklist reported the presence of 67 species; when supplemented with data from the Sudan Nature Guide project, this figure increases to 82 (unpublished data). Nonetheless, the actual species richness is likely underestimated, with projections suggesting the presence of more than 120 species (Schneider et al. 2020).

The genus *Paragomphus* Cowley, 1934 (family Gomphidae Rambur, 1842) is a large, predominantly African lineage which also extends into parts of Eurasia. Members of *Paragomphus* typically inhabit subtropical or tropical moist lowland forests and dry shrub lands, favouring open rivers, large water bodies, and shaded forest streams. They play key ecological roles in regulating insect populations and serve as bioindicators of freshwater ecosystem integrity. While several species are classified as Least Concern, others face pressures from habitat degradation, pollution, and climate change, rendering them Vulnerable or Endangered. Approximately 30 species are known from Africa, with additional species potentially yet to be described (Suhling and Martens 2007). Of these, 18 have been recorded in East Africa (Dijkstra and Clausnitzer 2014), of which five species have been recorded from Sudan. *Paragomphus pumilio* Rambur, 1842 is restricted to the Nile and occurs throughout its catchment from Egypt to Lake Turkana in north-eastern Kenya. *Paragomphus genei* Selys, 1841 and *P. sinaiticus* Morton, 1929 inhabit streams and springs in the Red Sea Hills, with recent records of *P. genei* from White Nile and Sennar states (Dumont and Martens 1984; Schneider et al. 2020). *Paragomphus sabicus* Pinhey, 1950 is known from a single record from Khartoum (Salah unpublished data). Reports of *P. cognatus* Rambur, 1842 from the Blue Nile and Atbarah rivers, originally published by Schneider et al. (2020), were later recognized, during the current review process, as misidentifications of female *Crenigomphus renei* Fraser, 1936, based on photographic evidence (K.-D.B. Dijkstra pers. comm.). A form closely resembling *P. lacustris* is described here as a new species: *Paragomphus alami*.

For comparative purposes, we refer to a subset of the African *Paragomphus* species that share key traits with *P. elpidius* Ris, 1921 as the *elpidius*-group. This informal grouping does not imply a formally resolved phylogenetic relationship. The key shared characters of this group include a notably short epiproct; parallel, blunt-tipped cerci; and green thorax. This group encompasses several African species, including *P. cataractae* Pinhey, 1963, *P. elpidius*, *P. lemperti* Dijkstra & Papazian, 2015, *P. nyasicus* Kimmins, 1955, and *P. lacustris* Karsch, 1890 which typically inhabit open water bodies and often large ones such as rivers and lakes (Dijkstra 2016).

## ﻿Materials and methods

### ﻿Study sites, sample collection, and documentation

Between January 2017 and March 2022, extensive fieldwork was conducted across multiple Sudanese states, including Khartoum, Sennar, Northern, River Nile, North Kordofan, White Nile, Gezira, Gadarif, and Kassala. In Khartoum State, comprehensive surveys were carried out along the White Nile, Blue Nile, and the Nile rivers throughout the year, encompassing ecological observations and individual counts.

Sampling localities:

1. 15°14'27"N, 32°27'03"E, 382 m a.s.l; Khartoum, Sudan, sample SNHM 1.582. Date: 23. Aug. 2019
2. 15°14'27"N, 32°27'03"E; alt. 382 m a.s.l.; Khartoum, Sudan, sample SNHM 1.583. Date: 23 Aug 2019.
3. 15°14'27"N, 32°27'03"E, 382 m a.s.l; Khartoum, Sudan, sample SNHM 1.584. Date:23. Aug. 2019
4. 15°14'27"N, 32°27'03"E, 382 m a.s.l; Khartoum, Sudan, sample SNHM 1.585. Date:23. Aug. 2019

Specimens were documented using several digital cameras (Canon D80, Power Shot SX60, A480, and Sony Cyber Shot DSC-HX1). Specimens were collected on three occasions: 23 August 2019, 15 September 2019, and 5 September 2022. Four specimens, obtained in 2019, were deposited at the Sudan Natural History Museum (**SNHM**) and assigned the museum numbers: SNHM 1.582, SNHM 1.583, SNHM 1.584, and SNHM 1.585. Collected specimens were dry-preserved in Odonata envelopes, then subsequently examined and photographed under a dissecting microscope. For molecular analysis, a single leg from each specimen was carefully detached and preserved in 100% ethanol for DNA extraction. High-resolution reference photographs of three original *Paragomphus lacustris* specimens from Lake Tanganyika archived at the Museum für Naturkunde, Berlin (Table 1) were obtained, examined, and morphologically compared with the Sudanese material. Additionally, comparative morphological assessments incorporated detailed reference images of *P. elpidius* from South Africa.

**Table 1.** Collection records and Natural History Universal Reference Identification system (NURI) accession codes for *Paragomphus lacustris* specimens from Lake Tanganyika, Museum für Naturkunde, Berlin (collector: Herrn Paul Reichard).

NURI	Sex	Remark
http://coll.mfn-berlin.de/u/85a0bb	Male	
http://coll.mfn-berlin.de/u/85a0ca	Male	Without head
http://coll.mfn-berlin.de/u/85a0c9	Female	

### ﻿Molecular analysis

Genomic DNA was extracted from two adult specimens using a modified salting-out protocol (Miller et al. 1988). The mitochondrial COI gene was amplified with universal primers LCO1490 and HCO2198 (Folmer et al. 1994) using i-Taq™ Premix and an annealing temperature of 47 °C. Amplicons (~750 bp) were verified by gel electrophoresis and sequenced commercially (BGI Genomics, China). Sequences were edited in BioEdit v. 7.0.5 (Hall 1999) and identified using BLAST® (Altschul et al. 1990). Laboratory work was conducted at the University of Khartoum under the DNA Barcode: A DNA-Based Registry for Some Animal Species in the Sudan project. The resulting COI sequences (Odo1, Odo2) were deposited in the Barcode Sudan Project (BOLD) under process IDs SDZBC009-21 and SDZBC010-21.

Twenty-eight COI sequences (570 bp) from *Paragomphus* spp. and related taxa were analysed to infer the phylogenetic position of *P. alami* (Table 2). A maximum-likelihood tree was generated in MEGA v. 12 (Kumar et al. 2024) using the Tamura 3-parameter (T92) model (Tamura 1992) with 1000 bootstrap replicates. The mean interspecific distance was computed manually after computing the pairwise distance in MEGA v. 12.

**Table 2.** COI sequences of *Paragomphus* species, *Crenigomphus renei*, and *Lamelligomphus ringens* used in phylogenetic analyses, including accession numbers, voucher codes, and geographic origin.

Species	Accession number (GenSeq)	Voucher number	Origin
*Paragomphus alami*	SDZBC009-21	SNHM 1.583	Sudan
*Paragomphus alami*	SDZBC010-21	SNHM 1.582	Sudan
*Paragomphus abnormis*	KU566251.1	RMNH.INS.559541	Angola
*Paragomphus abnormis*	KU566250.1	RMNH.INS.559541	Angola
*Paragomphus abnormis*	KU566248.1	RMNH.INS.508290	Angola
*Paragomphus abnormis*	KU566249.1	RMNH.INS.559580	Angola
*Paragomphus abnormis*	KU566247.1	RMNH.INS.559471	Angola
*Paragomphus abnormis*	KU566252.1	RMNH.INS.508787	Gabon
*Paragomphus elpidius*	KU566309.1	RMNH.INS.505333	DRC
*Paragomphus elpidius*	KINS2398.11	N/A	Kenya
*Paragomphus elpidius*	KINS2400-11	N/A	Kenya
*Paragomphus elpidius*	KINS2401.11	N/A	Kenya
*Paragomphus elpidius*	KINS2402-11	N/A	Kenya
*Paragomphus elpidius*	KINS2425-11	N/A	Kenya
*Paragomphus lemperti*	KU566324.1	RMNH.INS.503095	Sierra Leone
*Paragomphus lemperti*	KU566326.1	RMNH.INS.503130	Sierra Leone
*Paragomphus lemperti*	KU566327.1	RMNH.INS.503131	Sierra Leone
*Paragomphus lemperti*	KU566328.1	RMNH.INS.503132	Sierra Leone
*Paragomphus lemperti*	KU566325.1	RMNH.INS.504518	Liberia
*Paragomphus lemperti*	KU566329.1	RMNH.INS.504530	Liberia
*Paragomphus lemperti*	KU566323.1	RMNH.INS.506070	Liberia
*Lamelligomphus ringens*	OL663314.1	NSMK-IN-1603A0131	South Korea
*Paragomphus pumilio*	MW570959.1	14533	Sudan
*Crenigomphus renei*	KX891029.1	ODBOL-*1769*	Not available
*Paragomphus genei*	MT298593.1	MIB:ZPL:08474	Italy
*Paragomphus genei*	MT298592.1	MIB:ZPL:08475	Italy
*Paragomphus genei*	MT298591.1	MIB:ZPL:08476	Italy
*Paragomphus genei*	MN345045.1	USNM:ENT:00827258	Uganda

## ﻿Results

**Suborder Anisoptera Selys, 1854**

**Family Gomphidae Rambur, 1842**

### ﻿Genus *Paragomphus* Cowley, 1934

#### ﻿ Paragomphus alami sp. nov.

Taxon classificationAnimalia

D282A66D-4C9D-5363-AF9B-EA133B809DD4

https://zoobank.org/004B7FCE-9FAF-4506-9E8C-BA1DEEBB35CE

**Figure 1.**
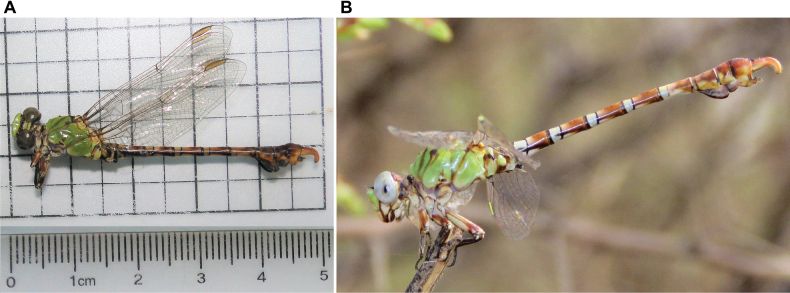
*Paragomphus alami* sp. nov. **A.** Male holotype (SNHM 1.582) from Jebel Aulia, Khartoum State, Sudan, collected 23 August 2019; **B.** Mature life male observed and photographed in the wild in Al-Sunt Forest, Khartoum State, Sudan, photographed 4 July 2022.

**Figure 2.**
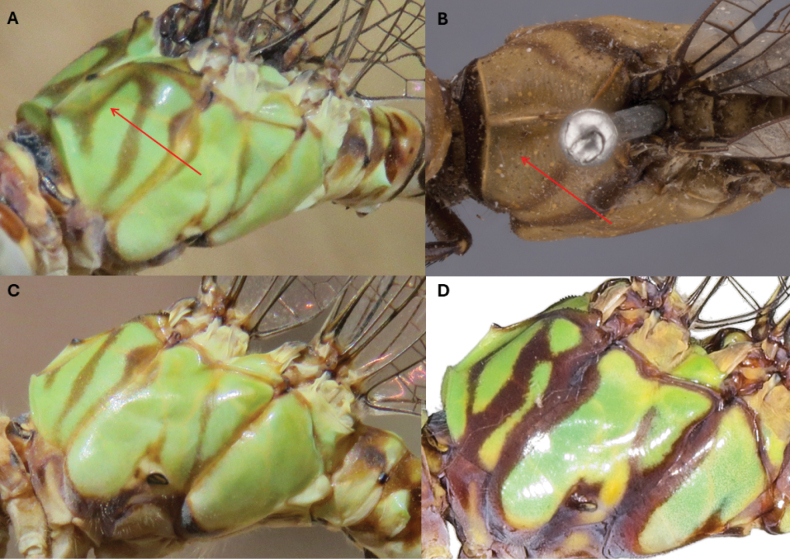
Thoracic coloration in *Paragomphus* species. **A, C.***P. alami* sp. nov.; **B.***P. lacustris*; **D.***P. elpidius*. Note the mesepisternal stripe of *P. alami* near the antealar sinus, extending anteriorly toward the head and dividing into two short branches that form Y shape (red arrow). Note also that the thoracic stripes in *P. alami* sp. nov. are fine and poorly defined, while in *P. elpidius* they are sharp, bold, and well defined.

**Figure 3.**
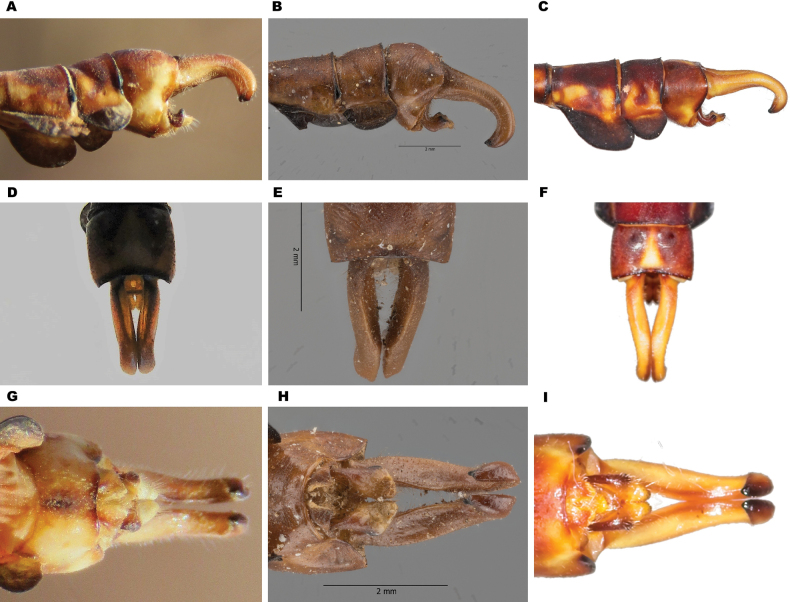
Male appendages of *Paragomphus* species. **A, D, G.***P. alami* sp. nov.; **B, E, H.***P. lacustris*; **C, F, I.***P. elpidius*. Shown in lateral (**A–C**), dorsal (**D–F**), and ventral (**G–I**) views. Note the short, thick cerci with a distinct ventral tooth in *P. alami*, the longer and more curved cerci in *P. lacustris*, and the slender cerci of *P. elpidius*.

**Figure 4.**
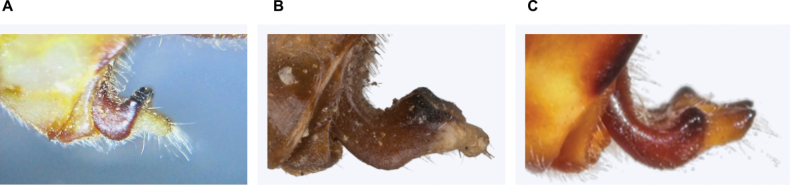
Differences in epiproct shape in *Paragomphus* males (in lateral view). **A.***P. alami* sp. nov.; **B.***P. lacustris*; **C.***P. elpidius*. The epiproct of *P. alami* sp. nov. is shorter than in *P. lacustris* and *P. elpidius*, its basal section especially being notably abbreviated, more strongly upcurved, and more sharply demarcated from the apical section. Also note the apical teeth of *P. elpidius*, which are absent in *P. alami* and *P. lacustris*.

**Figure 5.**
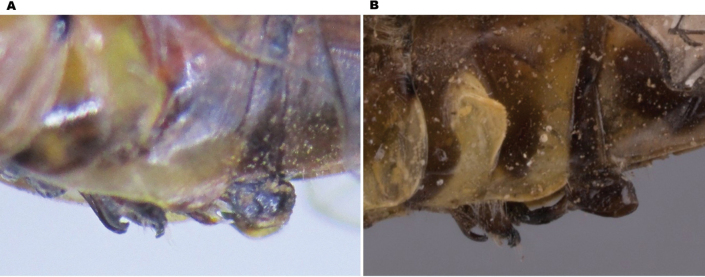
Secondary genitalia of *Paragomphus* species. **A.***P. alami* sp. nov.; **B.***P. lacustris*.

**Figure 6.**
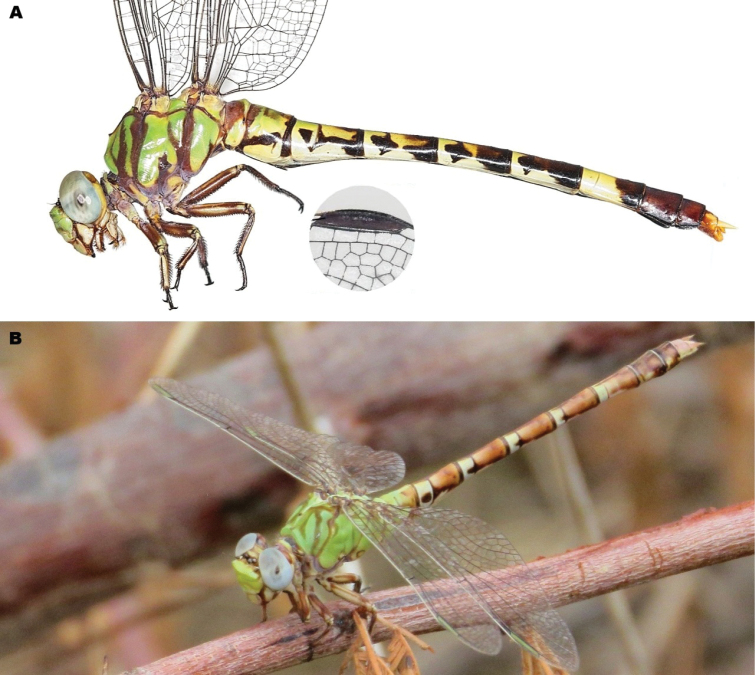
(mirrored). Comparison of female coloration and stripe patterns in *Paragomphus* species. **A.***P. elpidius*; **B.***P. alami* sp. nov. Note the small foliations on abdominal segments 8 and 9 in *P. elpidius*, which are absent in *P. alami* sp. nov. Note also that the thoracic stripes in *P. alami* sp. nov. are fine and poorly defined, while in *P. elpidius* they are sharp, bold, and well defined. Also, the pterostigma is yellowish green in *P. alami* sp. nov., while it is brown in *P. elpidius*. Photo credits: (**A**) John Wilkinson, (**B**) Mohamed Salah.

Figs 1, 2A, C, 3A, D, G, 4A, 5A, 6B

##### Type material.

***Holotype*.** Sudan • 1 ♂; Khartoum State, Jebel Aulia, north of Jebel Aulia Dam, floodplain on the western bank of the White Nile River, with *Vachellia* tree cover; large river with sandy bottom; 15°14'27"N, 32°27'03"E, 382 m a.s.l; 23. Aug. 2019; Mohamed Salah leg. Caught with an aerial net while resting in a *Vachellia* tree. BOLD ID: SDZBC010-21.COI-5P. Deposited in the Sudan Natural History Museum (SNHM 1.582) (Fig. 1A).

***Paratypes*.** Sudan • 2 ♀♀, 1 ♂; same locality as holotype: Khartoum State, Jebel Aulia, north of Jebel Aulia Dam, floodplain on the western bank of the White Nile River, with *Vachellia* tree cover; 15°14'27"N, 32°27'03"E; alt. 382 m a.s.l.; 23 Aug 2019 Mohamed Salah leg.; Deposited in the Sudan Natural History Museum (SNHM 1.583, SNHM 1.584, SNHM 1.585).

##### Other material.

Sudan • 2 ♂♂, 1 ♀; Khartoum State, Jebel Aulia, north of Jebel Aulia Dam, floodplain on the western bank of the White Nile River with *Vachellia* tree cover; 15°14'28"N, 32°28'04"E; alt. 380 m. a.s.l.; 5 Jul. 2022; Mohamed Salah leg.; preserved in the private collection of the first author (Mohamed Salah).

##### Diagnosis.

*Paragomphus alami* (Fig. 1B) closely resembles *P. lacustris* in the following: (a) habitus moderate size, hindwing length 24–25 mm; (b) labrum pale; (c) thorax yellowish-green, with fine, poorly defined brown stripes; (d) S8-9 with broad foliations; (e) cerci thick, with blunt apex ending in a black tooth and cerci parallel to each other to their end; (f) epiproct reduced to about one-third the length of the cerci. However, *P. alami* is readily distinguished from *P. lacustris* in the following: (1) pterostigma yellow but brown in *P. lacustris*; (2) mesepisternum bearing two stripes, one running parallel to the middorsal stripe, while the other is straight and situated near the antealar sinus, extending anteriorly toward the head and dividing apically into two short branches that form a distinct Y-shape (Fig. 2); (3) cerci shorter and less curved than in *P. lacustris*, which has longer cerci that are strongly curved toward the epiproct, especially when seen from the ventral view (Fig. 3); and (4) epiproct shorter than in *P. lacustris*, and its basal section, in lateral view, abbreviated, more strongly upcurved, and sharply demarcated from the apical section (Figs 3, 4).

*Paragomphus alami* is distinguished from *P. elpidius* and *P. lemperti* by the following: (1) pterostigma yellow but brown in *P. elpidius* and *P. lemperti*; (2) thoracic stripes fine and poorly defined but in *P. elpidius* and *P. lemperti* they are sharp, bold, and well defined; (3) cerci thick with blunt apex ending in a black tooth but in *P. elpidius* and *P. lemperti* they are slender and strongly curved and with a fairly blunt apex; and (4) epiproct shorter and its basal section, in lateral view, is abbreviated, more strongly upcurved, sharply demarcated from the apical section, and the epiproct’s two branches lack the sharp black tooth at their end, but in *P. elpidius* and *P. lemperti* the epiproct is relatively longer and the two branches terminate in a black sharp tooth.

##### Morphological description.

Total body length 49 mm, abdomen (excluding appendages) 36 mm, forewing length 27 mm, hindwing length 25.5 mm, forewing pterostigma 3.6 mm.

Eyes, in life, are pale blue; frons, anteclypeus, and postclypeus are yellowish green, with a median row of small, black spots on the postfrons. The vertex near the ocellus is light brown with yellow spots. The labrum, mandibles, and genae are uniformly pale.

The thorax is mostly yellowish green, with a thin brown metapleural stripe and a slightly broader brown middorsal stripe. The lower part of metepisternum below the metastigma is pale yellow. There are two fine brown stripes on mesepisternum: one runs parallel to middorsal stripe, and the other one straight and near the antealar sinus, extending anteriorly toward the head and dividing into two short branches at the end, where it forms a Y shape, a feature absent in *P. lacustris* (Fig. 2A, C). Both the metepimeron and mesepimeron near the legs have dark-brown ends. The base of the legs (coxa and trochanter) near the abdomen is creamy white. The femur is pale yellow, with a light-brown border, and the tibia and tarsus are dark brown to black. The wings are hyaline, lacking any tint or marking, and the pterostigma is yellow.

The abdomen, in general, is brown with white rings. Segment 1 is pale yellow to yellowish green, with a slight brownish tint posterodorsally near the tergite margins. Segment 2 bears a broad, central brown band, proximally yellowish-green and creamy white border at the genital lobes. Segments 3–7 are reddish brown, with pale intersegmental regions and distinct black apical rings. Segments 8 and 9 carry broad foliations; the one on segment 8 is broad, while the one on segment 9 is reduced. The upper halves of segments 8 and 9 are reddish brown, while the basal halves are pale. Segment 10 is apically reddish brown, with a pale terminal edge. The secondary genitalia are black (Fig. 5).

The caudal appendages are brown, and in dorsal view, the cerci are thick, with a blunt apex ending with black teeth; the cerci are parallel to one another to their end, resembling those of other *Paragomphus* species in the *elpidius*-group. The epiproct is short, and near the apex, there is a distinct gap between its branches, forming an eye-shaped opening (Fig. 3D). In lateral view, the cerci are short, thick, and with a blunt apex terminating with a black tooth; the epiproct is short (about one-third the length of the cerci) and shorter than the epiproct in both *P. lacustris* and *P. elpidius*. The basal section of the epiproct is notably abbreviated, more strongly upcurved, and sharply demarcated from the apical section. As a result, the epiproct’s dorsal processes appear as two relatively sharp black teeth, which lie between the epiproct’s base and tips. The apical section is comparatively short, and its two branches lack the terminal black teeth found in *P. elpidius*. In ventral view, the cerci are short, less curved, straighter toward the tips, and shorter than the *P. lacustris* cerci, which are longer and more curved toward the epiproct. The epiproct of *P. alami*, in ventral view, shows a distinct form that separates it from all other *elpidius*-group species; at the end, each branch is triangular (Fig. 3G).

The female is similar to male but lacks foliations on S8 and S9 (Fig. 6).

##### Etymology.

The species is named in honour of the late Sudanese scientist Dr Tigani Mohammed Hassan Alam, whose lifelong dedication to documenting and safeguarding Sudan’s wildlife left a profound impact on conservation efforts across the country. Although his contributions were not formally recorded in the academic literature, his influence endures through the generations of Sudanese youth he inspired to appreciate and protect the nation’s natural heritage.

##### Distribution, habitat, and biology.

*Paragomphus alami* was first recorded on 15 July 2017 in Sunt Forest (15°36'05"N, 32°29'59"E; 378 m a.s.l), a riparian woodland on the eastern bank of the White Nile River, which is dominated by *Vachellia nilotica*. At the time, the species was misidentified as *Paragomphus genei*.

**Figure 7.**
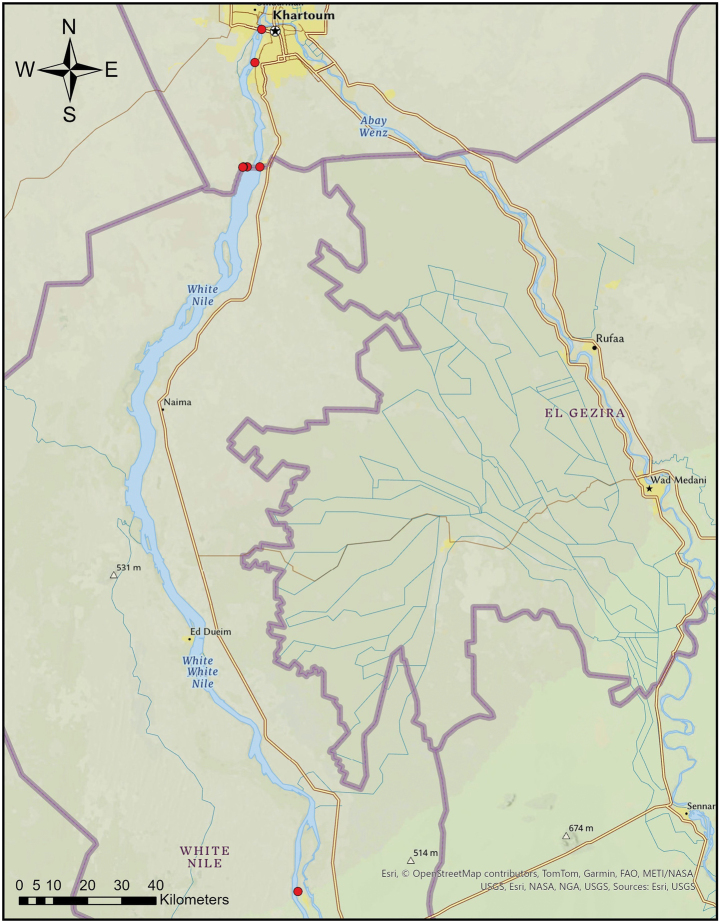
Map shows occurrence records of the *Paragomphus alami* sp. nov.

Between 2017 and 2023 it was recorded from five localities along the White Nile (Fig. 7). The species showed a strong seasonal occurrence in floodplain habitats along the White Nile. It was most abundant between June and September, particularly in areas with dense *Vachellia nilotica* cover such as at Jebel Aulia and Al-Dabbasin Bridge, where as many as 300–400 individuals were recorded during peak periods; they usually disappeared completely by October. In 2018, the *V. nilotica* tree cover at Al-Dabbasin Bridge was completely removed, leading to a sharp decline in the number of individuals observed, with only a single female recorded afterwards. This suggests a sensitivity to deforestation.

**Figure 8.**
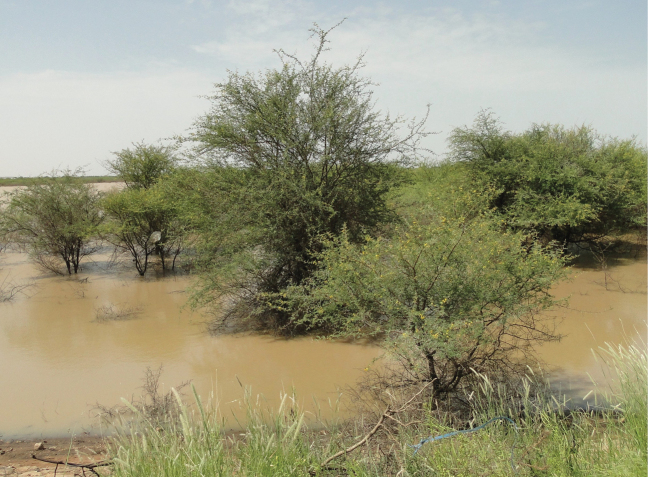
Floodplain on the western bank of the White Nile River, north of Jebel Aulia Dam, during the flooding season; photographed on 15 September 2019.

*Paragomphus alami* appears to be restricted to large, sandy rivers with well-developed riparian vegetation, particularly *Vachellia*-dominated floodplains (Fig. 8). The presence of adult stages appear to be associated with the density of *Vachellia* cover in surrounding floodplain habitats. Field observations suggest a flight period extending from early June to late September, with only a few individuals observed in early October. Adult emergence likely begins in early June. Adults are often seen resting on high branches in *Vachellia* trees, possibly due to advantageous camouflage. Individuals can occasionally be seen perched on branches and fallen trees closer to the ground. Mating has only been observed at dusk. These ecological associations are based solely on adult-stage observations; larval habitat preferences remain unknown.

### ﻿COI-based phylogeny and genetic divergence of *P. alami*

**Figure 9.**
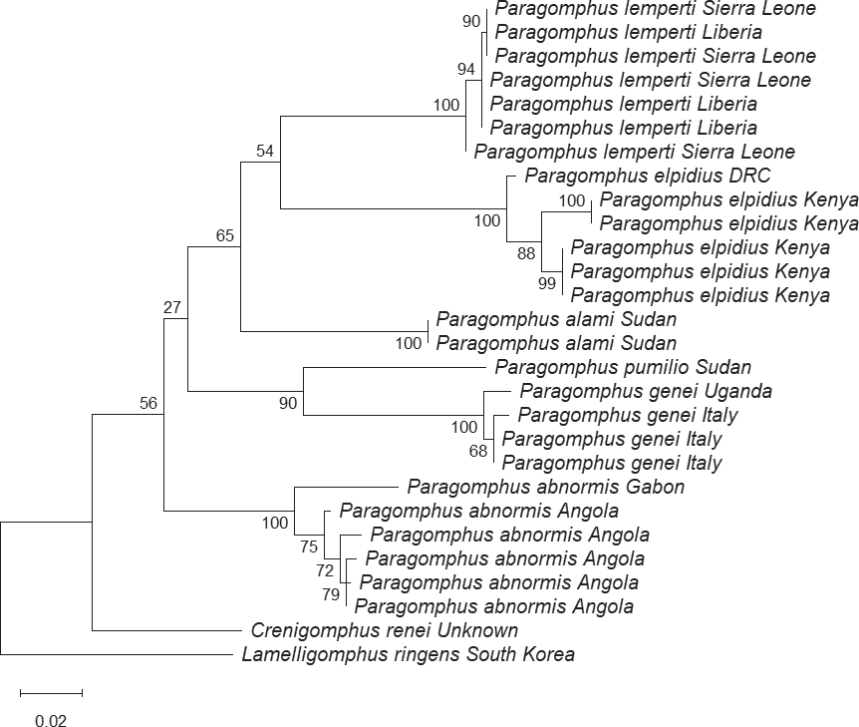
Maximum-likelihood phylogenetic tree, using T92 distance substitution model, based on 28 mitochondrial COI gene sequences (570 bp) of *Paragomphus* species from Africa and related sequences from GenBank. Bootstrap support values are shown at the nodes. The tree was rooted using *Lamelligomphus ringens* (South Korea) as an outgroup. The Sudanese *P. alami* cluster within distinct, well-supported clade (bootstrap support = 100%).

A maximum-likelihood (ML) phylogenetic tree was constructed using the Tamura 3-parameter (T92) substitution model using COI gene sequences from 28 specimens (Fig. 9). Bootstrap resampling was used to assess node support. *Paragomphus alami* from Sudan formed a distinct monophyletic lineage with strong statistical support (bootstrap = 100%), clearly separated from all other species included here. No intraspecific variation was detected within *P. alami*, corroborating its morphological distinctiveness as a new species. The mean interspecific distance between *P. alami* and other taxa was 12.34%, further supporting its status as a separate species.

### ﻿Key to males of the *elpidius*-group of *Paragomphus*

Modified from the key by Dijkstra and Clausnitzer 2014

**Table:** 

1	Cerci stout, only slightly longer than S10; epiproct short, at most half as long as S10 and strongly curved upwards or even into S10	***P. nyasicus***
–	Cerci slender, at least 1.5× as long as S10; epiproct longer, more than half as long as S10 and not so strongly curved upwards	**2**
2	Apical half of cerci very long and thin, with truncate apex bearing a long tooth; thorax uniformly bright green, at most with indiscernible brownish markings	***P. cataractae***
–	Cerci shorter, with blunt apex without a tooth or only a thick tooth; thorax yellow to green with brown to black markings	**3**
3	Cerci more slender with bluntly rounded tips without a prominent apical tooth; epiproct with its two branches bearing a sharp black tooth near their tips; dark thoracic stripes thick and well defined	**4**
–	Cerci thicker, their blunt tips ending with a thick black tooth; epiproct without apical black teeth; dark thoracic stripes fine and poorly defined	**5**
4	Curved section of cerci shorter and thicker; thoracic green and brown stripes are partly broken up and dead-ended	***P. elpidius***
–	Curved section of cerci longer and more slender; thoracic stripes are straight and continuous, extending unbroken between bases of legs and wings	***P. lemperti***
5	Cerci longer and curved towards epiproct; epiproct longer, its basal section in lateral view is not as sharply upcurved and smoothly transitions into the apical section, resulting in the two sections being indistinctly demarcated; pterostigma brown	***P. lacustris***
–	Cerci shorter and not curved towards epiproct; epiproct shorter, its basal section in lateral view being more strongly upcurved and sharply demarcated from the apical section; pterostigma yellowish	***P. alami***

## ﻿Discussion

### ﻿Taxonomic placement

*Paragomphus alami* exhibits morphological traits typical of a group characterized by a predominantly green thorax, parallel cerci, and a notably short epiproct, which is approximately one-third the length of the cerci. The morphology of the male appendages and the thoracic stripe pattern suggest a close relationship between *P. alami* and *P. lacustris*. DNA evidence indicates that *P. alami* is genetically close to *P. elpidius* and related to *P. lemperti*, as no DNA sequences are currently available for *P. lacustris* for direct comparison. This relationship is not unexpected, as *P. elpidius* is biogeographically the nearest known relative which is widespread in East and Southern Africa and recorded from Kenya and Uganda (Dijkstra 2016).

The considerable geographic separation between the confirmed records of *P. lacustris* and *P. alami* suggests long-term biogeographical isolation and independent evolutionary histories, with *P. alami* likely adapted to the more arid conditions of the Nile River system. The record from north-eastern Congo slightly narrows this gap (Clausnitzer et al. 2012), though the species’ identity of that material remains uncertain. Additionally, specimens from Uganda housed in the National Museums of Kenya and identified as *P. lacustris* (Njoroge et al. 2019) may in fact represent *P. alami*. This possibility raises the hypothesis that the common ancestor of both species may have originally occurred in Uganda, with *P. alami* subsequently diverging and expanding northward as a distinct evolutionary lineage.

### ﻿Habitat and ecology

The *elpidius*-group is ecologically associated with open running waters (especially larger rivers and lakes) throughout Africa (Dijkstra 2016). In Sudan, *P. alami* is restricted to the floodplain of the White Nile and is conspicuously absent from the nearby Blue Nile and Main Nile even though the confluence of the White and Blue Nile lies less than 1 km from its known habitat.

North of Malakal, the White Nile is characterized by a wide (3–4 km), shallow channel with slow and steady flow, lacking steep gradients, waterfalls, or strong currents. The main river channel spans 300–400 m, with a sandy bottom and an expansive floodplain with riparian woodland (Shahin 1985; Dumont 2009). These features provide favourable microhabitats for *P. alami* and contrast sharply with the Blue Nile, which possesses a narrow, deeply incised channel, strong current, and minimal floodplain development (Dumont 2009), conditions that seem unsuitable for *P. alami*.

Seasonal flooding in the White Nile north of the Jebel Aulia Dam occurs from July to October, peaking during Sudan’s rainy season. This inundation is caused by the Blue Nile’s high discharge (accounting for approximately 60–69% of the total Nile flow), which creates a temporary lake as the White Nile backs up. The floodwaters carry fertile sediment from the Ethiopian highlands (Shahin 1985), enriching the floodplain and further enhancing its ecological suitability for *P. alami*. Prior to the construction of the Jebel Aulia Dam, seasonal flooding extended across a broad area of the White Nile basin up to 40 km south of Khartoum. The dam’s completion in 1937 altered this hydrological regime, reducing floodplain connectivity (Tate et al. 2001) and possibly explaining the current rarity of *P. alami* further south.

The flying season is the period of emergence of adult odonates, which is influenced by the characteristics of their breeding habitat, whether permanent or temporary, the duration of larval development, and prevailing temperature conditions (Paulson 2019). *Paragomphus alami* adult flight period coincides with the annual flooding of the White Nile, typically spanning from early June to October.

As the White Nile represents a permanent freshwater system, larval development in *P. alami* is likely prolonged compared to species inhabiting ephemeral water bodies. In temperate zones, larval development in Gomphidae may extend up to six years, although most species complete their life cycle within three years (Corbet 1962). In tropical regions, emergence timing is generally governed by seasonal climatic patterns, particularly rainfall, with adult emergence frequently synchronized with the onset of the wet season. Despite limited understanding of the exact mechanisms underlying this regulation in tropical odonates (Paulson 2019), the emergence of *P. alami* during the flood season of the White Nile likely reflects an adaptation to favourable breeding conditions. This seasonal emergence ensures that adults encounter optimal habitat availability for mating and oviposition, particularly within inundated floodplain woodlands dominated by *Vachellia nilotica* tree cover. The marked seasonality observed in adult activity supports the species’ ecological reliance on hydrological cycles and underscores its sensitivity to changes in flooding regimes.

### ﻿Genetic distinctiveness

The molecular analyses strongly support *P. alami* as a genetically distinct and evolutionarily independent lineage. The ML phylogenetic tree, constructed under the best-fit substitution model, recovered a well-supported monophyletic clade comprising only the two *P. alami* sequences collected from Sudan. This clade was distinctly separated from all other *Paragomphus* species with a bootstrap value of 100% and a 12.34% interspecific distance, indicating robust confidence in its genetic isolation. No evidence of admixture or shared recent ancestry with related taxa was detected, consistent with the morphological data supporting the recognition of *P. alami* as a distinct species.

## ﻿Conclusions

Here we document the discovery and description of *Paragomphus alami*, a new dragonfly species currently only known from Sudan. This new species is distinguished by a unique combination of morphological characters that are strongly supported by molecular phylogenetic analyses. Morphological comparisons with closely related taxa, particularly *P. lacustris*, *P. elpidius*, and *P. lemperti*, confirmed the diagnostic distinctiveness of the Sudanese material. DNA barcoding and phylogenetic analyses further placed *P. alami* as a well-supported monophyletic lineage (bootstrap support = 100%), genetically divergent from all other species analysed. Ecological observations indicate that *P. alami* is restricted to floodplain habitats along the White Nile, where its abundance appears linked to *Vachellia nilotica* dominated riparian vegetation. Population fluctuations and recurrent local disappearances suggest sensitivity to deforestation and habitat alteration. The recognition of *P. alami* expands the known diversity of *Paragomphus* in Africa and underscores the urgent need for systematic surveys, molecular assessments, and habitat conservation to safeguard freshwater biodiversity in the Nile Basin.

## Supplementary Material

XML Treatment for Paragomphus alami

## ﻿Additional information

### Use of AI

No use of AI was reported.

### Author ORCIDs

Mohamed Salah https://orcid.org/0009-0004-8870-9248

Rania Baleela https://orcid.org/0000-0002-6914-9220

Esraa Yousif Ahmed https://orcid.org/0009-0004-7027-4709

Babiker Isam https://orcid.org/0009-0001-2609-1065

Almontasirbillah Abdalla https://orcid.org/0009-0000-9598-7554

Mai Masri https://orcid.org/0000-0002-9741-540X

Esra Elfaki https://orcid.org/0009-0006-4314-2211

### Data availability

All of the data that support the findings of this study are available in the main text.
